# Gut Microbiota Composition across Normal Range Prostate-Specific Antigen Levels

**DOI:** 10.3390/jpm11121381

**Published:** 2021-12-17

**Authors:** Han-Na Kim, Jae-Heon Kim, Yoosoo Chang, Dongmin Yang, Hyung-Lae Kim, Seungho Ryu

**Affiliations:** 1 Medical Research Institute, Kangbuk Samsung Hospital, Sungkyunkwan University School of Medicine, Seoul 03181, Korea; hanna147942@gmail.com; 2 Department of Clinical Research Design and Evaluation, SAIHST, Sungkyunkwan University, Seoul 06355, Korea; 3 Department of Urology, Soonchunhyang University Seoul Hospital, Soonchunhyang University Medical College, Seoul 04401, Korea; piacekjh@hanmail.net (J.-H.K.); dmyang93@gmail.com (D.Y.); 4 Center for Cohort Studies, Total Healthcare Center, Kangbuk Samsung Hospital, Sungkyunkwan University School of Medicine, Seoul 04514, Korea; 5 Department of Occupational and Environmental Medicine, Kangbuk Samsung Hospital, Sungkyunkwan University School of Medicine, Seoul 03181, Korea; 6 Department of Biochemistry, College of Medicine, Ewha Womans University, Seoul 07804, Korea; hyung@ewha.ac.kr

**Keywords:** prostate-specific antigen, androgen receptor activity, gut microbiota, 16S rRNA

## Abstract

Animal studies have shown the interaction between androgens and the gut microbiome directly and indirectly; however, limited evidence from human studies is available. To evaluate the association between prostate-specific antigen (PSA) levels within the normal range, reflective of androgen receptor activity, and the gut microbiota composition, a cross-sectional analysis was performed in 759 Korean men aged between 25 and 78 years with normal PSA levels of ≤4.0 ng/mL. We evaluated the biodiversity of gut microbiota as well as the taxonomic and functional signatures associated with PSA levels using 16S rRNA gene sequencing data. PSA levels within the normal range were categorized into three groups: lowest quartile (G1), interquartile range (G2, reference), and highest quartile (G3). The G3 group had higher microbial richness than the G2 group, although it was dominated by a few bacteria. An increase in Escherichia/Shigella abundance and a reduction in Megamonas abundance in the G3 group were also detected. A U-shaped relationship was observed between the three groups across most analyses, including biodiversity, taxonomic composition, and inferred pathways in the gut microbiota. This study showed different microbiota patterns across PSA levels within the normal range. Further studies are required to elucidate the role of microbiota in regulating PSA levels.

## 1. Introduction

The role of the gut microbiota in various clinical conditions has received considerable attention over the last decade. The gut microbiome is involved in the synthesis and regulation of numerous hormone-like chemical compounds; thus, it can play a functional role as an endocrine organ [1].

The androgen receptor (AR) target gene, known as prostate-specific antigen (PSA), has been recently reported as a possible marker of AR expression due to the correlation between PSA expression and the transcription of additional AR genes [2,3]. Our previous meta-analysis suggested that normal-range PSA levels could serve as a surrogate indicator of serum testosterone, and another prior study comprising a cohort of apparently healthy men showed that normal-range PSA levels, possibly reflective of androgen activity, could predict subclinical and clinical cardiovascular diseases [4,5].

According to the results of two animal studies, the gut microbiota can alter serum testosterone and sex hormone levels both directly and indirectly [6,7], while only limited human studies on this issue have been conducted. To date, only one cross-sectional study consisting of 31 men and 26 women using 16S rRNA gene sequencing has identified the correlation between serum testosterone levels and gut microbiota diversity and composition, but the sample size of the study was small and did not provide microbial functional pathways [8].

The present study investigated the association between PSA levels within the normal range and gut microbiota biodiversity and community structure. Additionally, specific bacterial taxa associated with PSA levels were investigated, and the functional capabilities of the microbial community were inferred based on 16S rRNA gene sequencing. These findings may serve as a foundation for further research on the involvement of the gut microbiome in regulating PSA levels, which reflect androgen activity.

## 2. Materials and Methods

### 2.1. Study Subjects and Group Definitions

**Figure 1:**
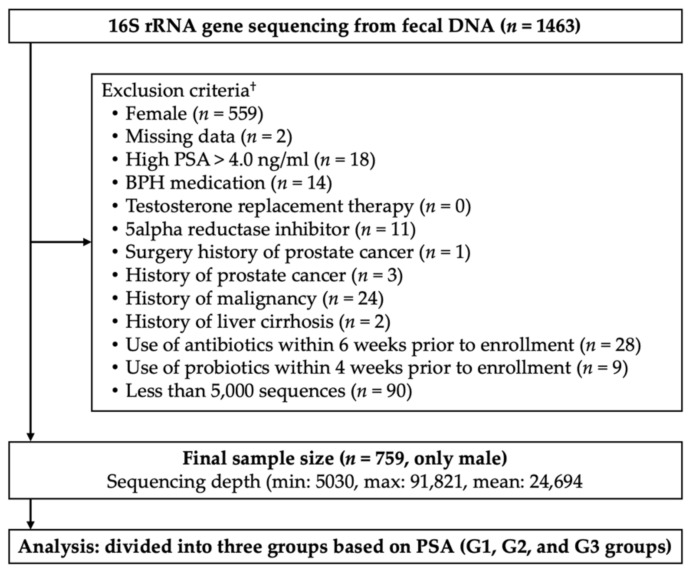
Enrollment of subjects. G1, lowest quartile (<25 percentile of the PSA range); G2, interquartile (from 25 percentile to 75 percentile of the PSA range); G3, highest quartile (>75 percentile of the PSA range). ^†^ Some subjects met several exclusion criteria.

In this study, we enrolled 1463 Korean men and women aged from 25 to 78 years who underwent a comprehensive annual or biennial physical examination between June and September 2014 at Kangbuk Samsung Hospital Healthcare Screening Center, Seoul, Republic of Korea. Among 1463 samples, subjects who met the following criteria were excluded: female (*n* = 559); missing data (*n* = 2); high prostate-specific antigen (PSA) > 4.0 ng/mL (*n* = 18); BPH medication (*n* = 14); testosterone replacement therapy (*n* = 0); 5alpha reductase inhibitor (*n* = 11); surgery history of prostate cancer (*n* = 1); prostate cancer (*n* = 3); history of malignancy (*n* = 24); history of liver cirrhosis (*n* = 2); use of antibiotics within 6 weeks prior to enrollment (*n* = 28); use of probiotics within 4 weeks prior to enrollment (*n* = 9); and samples with less than 5000 sequences (*n* = 90). Some individuals met more than one exclusion criterion, and a total of 759 participants were included in the final analysis (Figure 1).

Serum PSA levels were measured on the day of blood collection using an electrochemiluminescence immunoassay with the Modular E170 system (Roche Diagnostics, Tokyo, Japan). Subjects were categorized into three groups according to PSA level quartiles: lowest quartile (<25th percentile of the PSA range, G1), interquartile (from 25th percentile to 75th percentile of the PSA range, G2), and highest quartile (>75th percentile of the PSA range, G3).

The present study does not include any animal studies. All procedures involved in this study of human participants were in accordance with the ethical standards of the institutional research committee and with the 1964 Helsinki Declaration and its later amendments or comparable ethical standards. The present study was conducted according to a protocol approved by the Institutional Review Board of Kangbuk Samsung Hospital (KBSMB 2013-01-245-12 and 2020-09-003), and all participants provided written informed consents. Human participants’ names and other HIPAA identifiers were not used during the study process and were not included in all sections of the manuscript, including supplementary information.

### 2.2. Sample Collection, DNA Extraction, and 16S rRNA Gene Sequencing

Fresh fecal samples were collected into a sterile container from each volunteer enrolled in the study, immediately frozen at −20 °C after defecation, and stored at −70 °C for 24 h until further manipulation. Total DNA was extracted from stool samples within 1 month of storage using the MOBio PowerSoil DNA Isolation Kit (MO BIO Laboratories, Carlsbad, CA, USA) according to the manufacturer’s instructions. To amplify and sequence the V3-V4 hypervariable region of the 16S rRNA gene, specific fusion primers were used (Illumina, San Diego, CA, USA). Libraries were pooled for sequencing using the full complement of Nextera XT indices and sequenced on the Illumina MiSeq platform (Illumina, San Diego, CA, USA) according to the manufacturer’s instructions [9].

### 2.3. Microbial Profiling and Statistical Analysis

Demultiplexed sequences were processed using DADA2, which is a plugin of the QIIME2 package (version 2017.11, https://qiime2.org, accessed on 10 November 2017) [10], and the low-quality regions of the sequences and chimeras were removed. Amplicon sequence variants (ASVs) were generated by denoising with DADA2 and regarded as 100% operational taxonomic units (OTUs). A feature table containing the counts of each unique sequence in each sample was constructed. Features that were present in only one sample were filtered considering that they did not represent real biological diversity but were PCR or sequencing errors.

Microbial diversity analyses were performed with rarefied data of 5030 sequences per sample by random subsampling to minimize differences among samples due to sequencing depth in the QIIME2 package (version 2020.8, https://qiime2.org, accessed on 7 September 2020). The biodiversity of the samples, α-diversity, was calculated using the number of ASVs observed in each sample, Shannon index accounting for both evenness and richness, Faith’s phylogenetic diversity (PD) [11], and Pielou’s evenness. The Kruskal–Wallis test was used as a non-parametric statistical test to compare the different groups because some of the variables analyzed were not normally distributed. The average sequencing depth per sample was 24,694 reads, ranging from 5030 to 91,821 reads. The 3433 features remained in 759 subjects after contingency-based filtering of features. After rarefying the feature tables to 5030 reads per sample, rarefaction curves showed that all groups tended to plateau, indicating that the biodiversity was adequately covered with the applied sequencing depth (Figure S1).

The dissimilarity between samples, β-diversity, was calculated using the UniFrac distance [12] to estimate dissimilarity among group members by incorporating the phylogenetic distances between ASVs. Unweighted and weighted UniFrac distances were calculated to determine the presence/absence and abundance of ASVs, respectively. Non-phylogenetic β-diversity indices, such as Bray–Curtis dissimilarities [13], were also used for the abundance data. Pairwise permutational multivariate analysis of variance (PERMANOVA) with 999 random permutations was used to test the significance of differences between groups. Microbial community composition was depicted as principal coordinate analysis (PCoA) plots. All the tests were corrected for multiple tests. Basic statistical analyses were performed using RStudio (version 1.3.1073, Boston, MA, USA), and plots of microbial diversity were depicted using the ggplot2 package (version 3.3.2) in RStudio.

For taxonomic structure analysis, taxonomy was assigned to ASVs using a pre-trained naïve Bayes classifier and the q2-feature-classifier plugin against the Silva database (version r138.1) of the 16S rRNA sequence database in the QIIME2 package (version 2020.8, https://qiime2.org, accessed on 7 September 2020). To investigate significant differences in the relative abundance of any taxa from phylum to genus levels between groups, two statistical tools, analysis of composition of microbiomes (ANCOM, v2.1) [14] and generalized linear models implemented in multivariate association with linear models (MaAsLin2), which are R packages (R version 4.0.2) were used. After adjusting for age and BMI, we compared the abundance of taxa between pairwise groups among G1, G2, and G3 groups. ANCOM compares the relative abundance of taxa among groups by the log ratio of the abundance of each taxon to the abundance of all the remaining taxa, one at a time. The final significance was expressed in the empirical distribution of W at each taxonomic level. We used the taxa-wise FDR option and set the significance level to FDR < 0.05 to generate W statistics and a threshold of 0.8 for declaring an association as significant. MaAsLin2 set the G2 group as the reference and compared the G1 and G2 groups. The coefficient values and *q*-values that passed the significance threshold (Benjamini–Hochberg false discovery rate, FDR *q*-value < 0.05) were represented by the MaAsLin2 analyses. Linear Discriminant Analysis Effect Size (LEfSe) was also conducted to detect potential PSA-specific bacterial markers [15]. Only taxa with an LDA score (log10) greater than 3 (*p*-value < 0.05) were considered significantly enriched.

For functional inferences of the microbial community, Phylogenetic Investigation of Communities by Reconstruction of Unobserved States 2 (PICRUSt2) (v2.2.0-b) [16] was conducted with ASVs according to the instructions published at https://github.com/picrust/picrust2/wiki (accessed on 24 September 2020). We generated metabolic pathway database (Metacyc) pathway abundance predictions from Enzyme Classification numbers (EC numbers, as of 21 January 2016)-based gene family predictions [17]. Predicted functional pathways were compared among the groups using statistical analysis of taxonomic and functional profiles (STAMP) version 2.1.3 [18]. Statistical differences in the pathways were tested using Welch’s *t*-test with a Benjamini–Hochberg FDR correction (*q* < 0.05) to adjust *p*-values for multiple testing.

## 3. Results

### 3.1. Subject Demographics

Based on PSA levels, 759 men (mean age: 41.77 years) were categorized into the lowest quartile (G1, *n* = 189), interquartile (G2, *n* = 379), and highest quartile (G3, *n* = 191) (Table 1). Mean PSA was 0.44, 0.83, and 1.77 in the G1, G2, and G3 groups, respectively. PSA levels were positively associated with age and HDL-C and negatively associated with BMI, waist circumference, systolic BP, and triglycerides. No significant differences were observed in the prevalence of hypertension, diabetes, and obesity among the three groups. There was no significant difference in nutritional intake among the three groups (Table S1).

### 3.2. Comparison Microbial Diversity of among the PSA Groups

**Figure 2:**
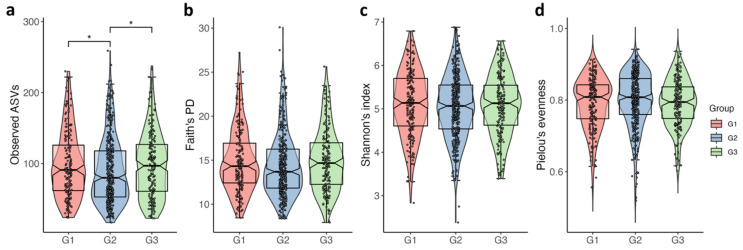
Violin plots showing the distribution of alpha diversity as measured by (**a**) observed ASVs, (**b**) Faith’s phylogenetic diversity (PD), (**c**) Shannon’s index, and (**d**) Pielou’s evenness, among the PSA groups. Statistics were calculated using a pair-wise Kruskal–Wallis test. * *q* < 0.05. The notched boxes indicate the interquartile range (IQR) of the 25th to 75th percentiles. The median value is shown as a line within the box, and the whiskers extend to the most extreme value within 1.5 × IQR. Each point represents an individual subject.

With regard to α-diversity, a U-shaped relationship was noted between PSA categories and microbial biodiversity (Figure 2). The G2 group had significantly lower microbial biodiversity than the G1 and G3 groups. The number of ASVs observed in each sample was significantly higher in the G1 and G3 groups than in the G2 group (Figure 2a, G1 vs. G2, *q* = 0.048; G2 vs. G3, *q* = 0.048). Phylogenetic biodiversity, Faith’s PD, was higher in the G3 group than G2, but did not pass the significance threshold for multiple comparisons (Figure 2b, *p* = 0.026, *q* = 0.079). In contrast, the G3 group had lower evenness than the G2 group, indicating that a few bacteria were predominant in the gut of the G3 group (Pielou’s evenness, *p* = 0.024, *q* = 0.072). The evenness showed an inverted U-shaped distribution among the three groups (Figure 2d). Overall, the G3 group had high microbial richness and low evenness; however, this finding did not reach statistical significance (*q* < 0.05) in the pair-wise comparison, except for the observed features, although they showed an unadjusted *p*-value < 0.05.

**Figure 3:**
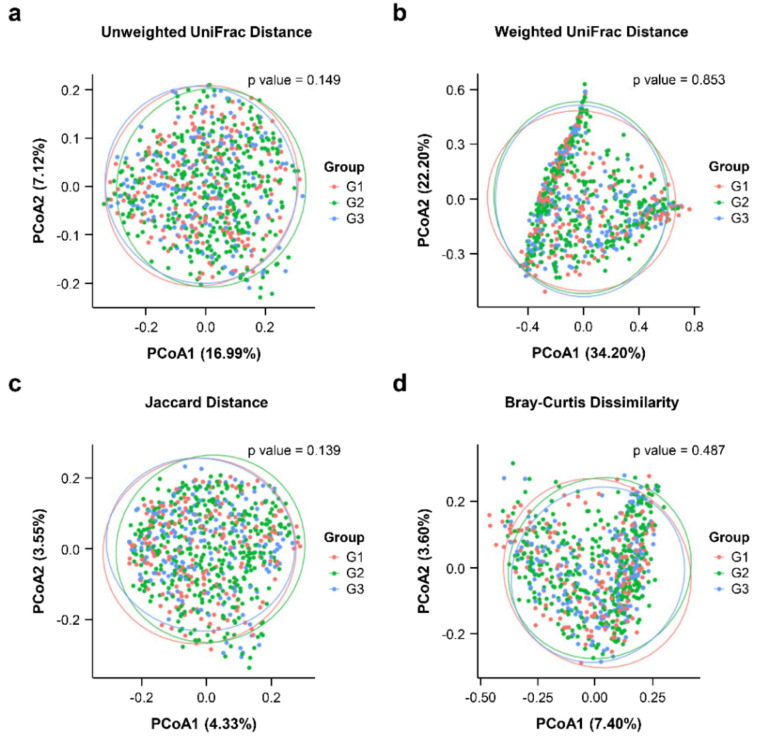
Beta-diversity of microbial taxa. Principal coordinate analysis (PCoA) plots representing the β-diversity for (**a**) unweighted UniFrac distance, (**b**) weighted UniFrac distance, (**c**) Jaccard distance, and (**d**) Bray–Curtis dissimilarity comparing subjects by PSA. Each point represents an individual subject. Statistics were calculated using pairwise PERMANOVA with 999 permutations. Ellipses represent the 95% confidence interval for each group.

In addition to α-diversity, the unweighted and weighted UniFrac distance, Jaccard dissimilarity, and Bray–Curtis dissimilarity matrix were analyzed to compare similarities among gut microbial communities (β-diversity). No significant differences were observed in the β-diversity based on pairwise PERMANOVA (Table S2). Moreover, no significant clustering was noted in the PCoA plots of all indices for β-diversity; however, G1 and G3 were clustered closer together than G2 (Figure 3).

### 3.3. Fecal Bacterial Community Abundance among the PSA Groups

A total of 14 phyla were identified, considering all ASVs defined. Overall, the most abundant taxa represented were Bacteroidetes (51.71%), Firmicutes (42.92%), and Proteobacteria (3.38%), whereas Actinobacteria, Fusobacteria, Verrucomicrobiota, and Desulfobacterota constituted minor phyla, each contributing less than 1% of the total sequences.

**Figure 4:**
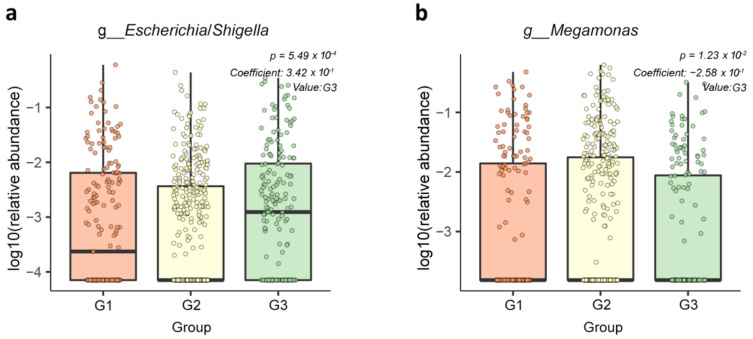
Relative abundance of genera associated with PSA. (**a**) The genus Escherichia/Shigella was more abundant in the G3 group than in the G2 group. (**b**) The genus Megamonas showed lower abundance in the G3 group than in the G2 group. Plots were depicted using MaAsLin2. MaAsLin analyses were performed using default options with adjusting for age and BMI.

To investigate differences in the taxonomic abundance between PSA groups, ANCOM was first used, as it incorporates the compositionality of microbiome data, has a low false-positive rate, and allows covariate adjustment [14,19]. The G2 group was used as a reference based on microbial diversity results. Regarding the phylum to family level, no statistical differences were revealed among the three PSA groups. Two genera with a normalized W of at least 0.8 were identified, indicating a significant change in these genera compared to the rest of the genera in the community in at least 80% of the comparisons (Table S3). Of the 255 genera, Escherichia/Shigella and Megamonas were statistically significant between G2 and G3 (W = 221 and W = 212, respectively). There was no significant difference in taxonomic abundance between the G2 and G1 groups. These results were confirmed with the MaAsLin (*p* = 5.49 × 10^−4^ for Escherichia/Shigella; *p* = 1.23 × 10^−2^ for Megamonas). The abundance of Escherichia/Shigella was 1.41 times higher in G3 than in the G2 group after adjusting age and BMI (coefficient = 0.34, exponentiated coefficient = 1.41, Figure 4a), while the abundance of Megamonas was lower in G3 than the G2 group (coefficient = −0.26, exponentiated coefficient = 0.77, Figure 4b).

**Figure 5:**
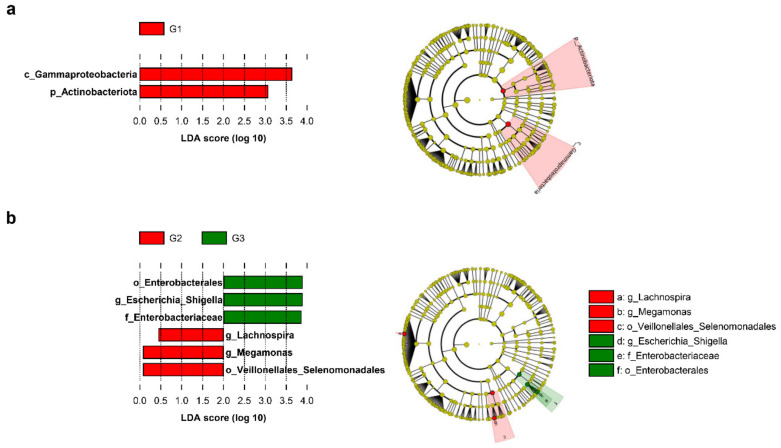
Linear discriminant analysis effect size (LEfSe) analysis of microbial abundance among PSA groups. A forest plot and Cladogram showing taxa that were significantly differentially abundant between (**a**) the G1 (red) and G2 (green) groups and (**b**) the G2 (red) and G3 (green) groups, respectively, as determined using the Kruskal–Wallis test. LDA score (effect size) indicating significant differences in bacterial taxa (LDA score >3.0; alpha value *p* < 0.05). Cladogram generated using the LEfSe method showing the phylogenetic distribution of microbes associated where taxonomic levels of phylum, class, and order are labeled, whereas the family and genus are abbreviated. Plots were depicted using the LEfSe of the Galaxy of the Huttenhower lab.

To further analyze the fecal microbiota pattern between the G2 and G3 groups, a linear discriminant analysis coupled with LEfSe was performed. The forest plot (Figure 5a) and the cladogram (Figure 5b) were generated from the LEfSe analysis, which showed the most differentially abundant taxa enriched in microbiota with green for the G3 group and red for the G2 group. The significance of Escherichia/Shigella in the G3 group was confirmed by the high LDA score in LEfSe. Additionally, the order Enterobacterales and the family Enterobacteriaceae, which were upper taxa of Escherichia/Shigella were also significantly enriched in the G3 group (LDA > 3, Figure 5). Megamonas belonged to the order Veillonellales/Selenomonadales, and the genus Lachnospira were significantly enriched in the G2 group in comparison with the G3 group.

### 3.4. Predicted Functional Pathways

**Figure 6:**
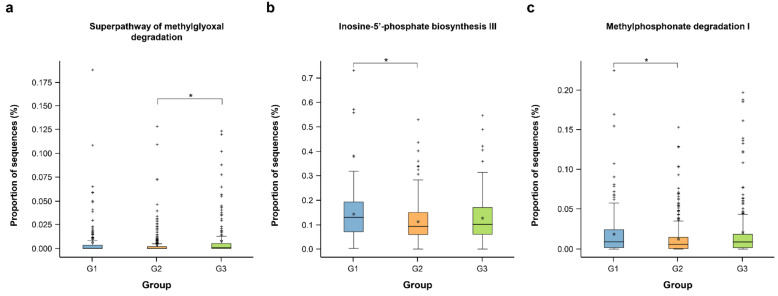
Predicted functional pathways were differentially abundant among the PSA groups. Pathway Database (Metacyc) pathways using PICRUSt2 analysis. (**a**) Superpathway of methylglyoxal degradation was more abundant in the G3 group than in the G2 group (*q* = 0.032), (**b**) inosine 5′-phosphate biosynthesis III (*q* = 0.037), and (**c**) methylphosphonate degradation I (*q* = 0.041) were more abundant in the G1 group than in the G2 group. The box plots denote the top quartile, median, and bottom quartile, and the white stars represent the average value. All differences were analyzed using MaAsLin2 with adjusting for age and sex. The Benjamini–Hochberg FDR method was used to correct multiple comparisons. * *q* < 0.05.

PICRUSt2 was performed to explore the functional profiles of gut microbiota associated with PSA. Using the Metacyc database, the superpathway of methylglyoxal degradation was significantly more abundant in G3 than in G2 (Figure 6a). Two pathways, inosine 5′-phosphate biosynthesis III and methylphosphonate degradation I, were significantly more abundant in G1 than in G2 (Figure 6b,c, respectively).

## 4. Discussion

Evidence from animal studies has demonstrated that steroid metabolism and sex hormone levels can be affected by gut microbes [1,20]; however, human studies on this topic are limited, and the underlying mechanisms have not been fully elucidated. This study sought to examine the composition of gut microbiota and the inferred microbial functions across different PSA levels, possibly reflective of androgen activity. Recently, the gut microbiota has received attention as a major regulator of androgen metabolism in the intestine, affecting androgen levels [21]. Correlation between androgen activity and the gut microbiome has been reported in only one study with a small sample size of 31 men and 26 women [8].

In the current study, changes in the biodiversity and composition of the gut microbiota induced by PSA within a normal range were evaluated. The highest quartile PSA (G3) group had more diverse bacteria than the interquartile PSA (G2) group, but the intestines of the highest quartile group were likely to be dominated by a few bacteria.

Shin et al. reported no difference in the bacterial community between males and females, but men with high levels of testosterone (>4.55 ng/mL) had more gut microbial diversity and evenness compared to those with medium levels of testosterone [8]. Biodiversity showed a U-shaped trend in the previous study, similar to the alpha diversity results in the current study, although PSA levels in all the participants in this study were within the normal range.

Interestingly, a U-shaped relationship between normal range PSA levels and the gut microbiota was identified in most of the analyses, including biodiversity, taxonomic composition, and pathway analyses based on 16S data. The lowest and highest quartile groups of normal range PSA showed similar characteristics of gut microbiota. Some studies have shown that gut microbiota can alter serum testosterone and sex hormone levels [6,7], while androgen exposure has been reported to affect gut microbiota dysbiosis [22,23]. Thus, the gut microbiota and androgen levels might affect each other. The cross-sectional design of this study could not determine the temporal directionality of the association between PSA levels and gut microbiota. Further longitudinal studies with repeated measurements of PSA, sex hormones, and gut microbiota may help better understand their interrelationships.

Research on mouse models revealed that the ratio of Firmicutes to Bacteroidetes in the gut microbiome was elevated when hypogonadism was induced [24]. Recent studies have also reported that endogenous and exogenous testosterone are correlated with fluctuations in the gut microbiota composition [20,24]. Firmicutes and Bacteroidetes were also the most dominant in the participants tested in this study, but were not significantly different among the PSA groups, indicating that the two phyla are not associated with PSA levels (Figure S2).

An increase in Escherichia/Shigella abundance and a reduction in Megamonas abundance was detected in the highest PSA quartile group (G3). Both Escherichia and Shigella are Gram-negative bacteria but cannot be distinguished by their 16S rRNA gene sequence [25], and as such, have been grouped together as one genus in the Silva DB. It has been reported that sex steroid hormones act as substrates of E. coli multidrug efflux (MDE) pumps, which are important factors in resistance against bile acids [26]. Recently, a correlation analysis between serum metabolites and sex-biased bacteria revealed that the serum concentration of androgen-related metabolites was positively correlated with Bulleidia and Escherichia [27]. In this study, the highest PSA group had a higher abundance of Escherichia/Shigella than the G2 group. This might be due to the role of Escherichia in androgen-related metabolites, in which beta-gluconidase released from Escherichia separates glucuronide and androgen conjugates, increasing the reabsorption of free androgens [28]. Another study performed using a mouse model also supports the finding that elevated amounts of testosterone correlate with conventional microbiota colonization and Escherichia/Shigella abundance [29]. Although they did not investigate the correlation with testosterone level, Singh et al. [30], in a human study, reported sexual differences in the overall gut microbiota and that Escherichia was more abundant in males than in females. This finding supports a model wherein Escherichia in the gut might increase host testosterone by regulating androgen metabolism, or that in which host testosterone can increase the abundance of Escherichia in the gut.

In a recent study, the genera Megamonas, Actinobacter, Dorea, and Ruminococcus were found to be positively correlated with serum testosterone concentrations in men, based on 16S rRNA sequencing results [8]. In another study on sexual differences in gut microbiota, the genera Megamonas, Prevotella, Fusobacterium, and Megasphaera were found to be more abundant in males than in females [31]. In contrast, in our study, the abundance of Megamonas was lower in the group with high PSA level (G3) than in that with intermediate PSA level (G2); moreover, we observed no association between PSA level and other previously reported genera [8,31]. Given the differential androgen measurements and smaller sample size in this study compared with those in the abovementioned previous reports [8,31], the potential role of Megamonas in androgen metabolism warrants further studies.

We further found that the taxa related to the methylglyoxal (MGO) degradation pathway were more enriched in the G3 than in the G2 group. MGO is a toxic, physiological reactive metabolite of glucose and a potent precursor of cytotoxic advanced glycation end products (AGEs) [32]. Recently, ovarian tissue has been identified as a target of excessive AGE deposition, which has been associated with either a high-AGE diet in experimental animals or hyperandrogenic disorders such as polycystic ovarian syndrome in humans [33]. MGO has further been shown to be capable of inducing apoptosis in prostatic cancer PC-3 cells, thus acting as a potential prostate cancer treatment agent [34]. Further research on the clinical implications of the MGO degradation pathway at high PSA levels is therefore needed.

Several limitations in the current work must be noted. First is the failure to specify the levels of direct androgen activity or androgens, including serum testosterone. Nevertheless, PSA has been demonstrated in earlier studies to have the potential as a surrogate marker for androgen activity [2,3]. Prioritizing androgen activity over sex hormones is justified, given that androgen activity, which could be construed as a receptor of androgen expression, could be of clinical significance. Second, the correlation between obesity and the gut microbiome was not addressed. Male individuals are more prone to morbidities associated with obesity because of their tendency to develop an apple-shaped constitution with significant deposits of adipose tissue around the viscera [35]. Nevertheless, this is the largest population-based study that demonstrates the association between the gut microbiota and PSA within the reference range, showing differences in biodiversity and taxonomic composition across different PSA concentrations.

## 5. Conclusions

An exclusive correlation between PSA and distinctive microbiota patterns had not yet been reported. This study showed different microbiota patterns across PSA levels within the normal range. Further longitudinal and mechanistic studies are required to elucidate the role of microbiota in PSA levels or vice versa.

## Figures and Tables

**Table 1:** Mean values (standard deviation) and the number (proportion) of baseline characteristics of the study participants by PSA.

Characteristics	Overall	Groups by PAS, ng/mL (Minimum–Maximum)			*p* for Trend
		G1 (<25 Percentile) (0.13–0.58)	G2 (Interquartile) (0.58–1.16)	G3 (>75 Percentile) (1.17–3.76)	
Number (male)	759 (100)	189 (100)	379 (100)	191 (100)	
Age (year)	41.77 (±8.95)	41.31 (±8.92)	40.67 (±8.15)	44.38 (±9.95)	<0.001
BMI (kg/m^2^)	24.65 (±2.79)	25.06 (±3.03)	24.72 (±2.78)	24.10 (±2.44)	<0.001
Waist circumference (cm)	86.24 (±7.46)	86.91 (±8.02)	86.29 (±7.30)	85.14 (±7.11)	0.021
Systolic BP (mmHg)	114.10 (±12.29)	115.02 (±13.67)	114.43 (±11.68)	112.53 (±11.94)	0.048
Diastolic BP (mmHg)	74.37 (±9.41)	74.58 (±10.19)	74.39 (±9.09)	74.10 (±9.27)	0.625
Current smoker	213 (28.06)	55 (29.10)	110 (29.02)	48 (25.13)	0.441
Alcohol intake ^1^	265 (34.91)	67 (35.45)	122 (32.19)	76 (39.79)	0.257
PSA (ng/mL)	0.97 (±0.58)	0.44 (±0.10)	0.83 (±0.15)	1.77 (±0.58)	<0.001
Glucose (mg/dL)	99.72 (±20.20)	102.24 (±25.65)	97.97 (±16.45)	100.70 (±20.63)	0.463
Total cholesterol (mg/dL)	200.30 (±34.65)	201.47 (±35.51)	200.00 (±34.99)	199.76 (±33.23)	0.632
LDL-C(mg/dL)	123.75 (±30.51)	126.20 (±30.86)	123.03 (±30.25)	122.68 (±30.69)	0.257
HDL-C (mg/dL)	52.88 (±12.82)	50.16 (±10.78)	53.60 (±13.54)	54.15 (±12.86)	0.002
Triglycerides (mg/dL)	118.00 (±78.00)	125.00 (±77.00)	117.00 (±78.00)	116.00 (±75.50)	0.030
hsCRP (mg/dL)	0.73 (±1.63)	0.65 (±1.02)	0.69 (±1.53)	0.87 (±2.20)	0.178
Hypertension	142 (18.71)	35 (18.52)	65 (17.15)	42 (21.99)	0.384
Diabetes	75 (9.88)	21 (11.11)	32 (8.44)	22 (11.52)	0.890
Obesity	551 (27.40)	58 (30.69)	103 (27.18)	47 (24.61)	0.195
Metabolic syndrome	134 (17.65)	39 (10.63)	64 (16.89)	31 (16.23)	0.262

Data are presented as *n* (%), mean (±SD), or median (interquartile range). PSA—prostate-specific antigen; BMI—body mass index; BP—blood pressure; LDL-C—low-density lipoprotein cholesterol; HDL-C—high-density lipoprotein cholesterol; hsCRP—high-sensitivity C-reactive protein. ^1^ ≥20 g of ethanol per day.

## Data Availability

Raw sequencing data of the 16S rRNA gene obtained in this study have been deposited in the public repository Clinical and Omics Data Archives (CODA) of the Korea National Institute of Health (accession number R000635; http://coda.nih.go.kr/coda/coda/search/omics/genome/selectSearchOmicsGenomePop/R000635.do, accessed on 31 March 2018).

## Supplementary Materials

The following are available online at https://www.mdpi.com/article/10.3390/jpm11121381/s1, Figure S1: Rarefaction plots based on alpha diversity metrics, Figure S2: Mean relative abundance of Firmicutes and Bacteroidetes among PSA groups, Table S1: Nutrient characteristics of study participants according to PSA, Table S2: Statistical significance among three groups by PSA using distance matrices for beta-diversity, Table S3: Comparison of taxonomic composition of gut microbiota among PSA groups.
