# Supplementary material for: Gut Microbiota Composition across Normal Range Prostate-Specific Antigen Levels
Source: J Pers Med. 2021 Dec 17;11(12):1381. doi: 10.3390/jpm11121381 (PMC8703440; doi:10.3390/jpm11121381)
Supplement: Supplementary file 1 [file jpm-11-01381-s001.zip › jpm-1503066-supplementary.pdf]

Supplementary Materials

Gut Microbiota Composition across Normal Range Prostate-Specific Antigen Levels

Han-Na Kim [1,2,†], Jae Heon Kim [3,†], Yoosoo Chang [2,4,5,*], Dongmin Yang [3], Hyung-Lae Kim [6] and Seungho Ryu [2,4,5,*]

1 Medical Research Institute, Kangbuk Samsung Hospital, Sungkyunkwan University School of Medicine, Seoul 03181, Korea; hanna147942@gmail.com
2 Department of Clinical Research Design and Evaluation, SAIHST, Sungkyunkwan University, Seoul 06355, Korea
3 Department of Urology, Soonchunhyang University Seoul Hospital, Soonchunhyang University Medical College, Seoul 04401, Korea; piacekjh@hanmail.net (J.-H.K.); dmyang93@gmail.com (D.Y.)
4 Center for Cohort Studies, Total Healthcare Center, Kangbuk Samsung Hospital, Sungkyunkwan University School of Medicine, Seoul 04514, Korea
5 Department of Occupational and Environmental Medicine, Kangbuk Samsung Hospital, Sungkyunkwan University School of Medicine, Seoul 03181, Korea
6 Department of Biochemistry, College of Medicine, Ewha Womans University, Seoul 07804, Korea; hyung@ewha.ac.kr
* Correspondence: yoosoo.chang@gmail.com (Y.C.); sh703.yoo@gmail.com (S.R.); Tel.: +82-2-2001-5139 (Y.C.); +82-2-2001-5137 (S.R.)
† These authors contributed equally to this work.

.

Table S1. Nutrient characteristics of study participants according to PSA.

Nutrients	G1 (n = 146)	G2 (n = 297)	G3 (n = 153)	p value
Total energy, kcal/day	1,531.4 ± 709.3	1,475.7 ± 596.3	1,472.2 ± 609.9	0.636
Total carbohydrate, g/day	260.7 ± 128.8	246.1 ± 99.6	247.5 ± 100.4	0.383
Total fat, g/day	29.3 ± 19.6	29.8 ± 19.2	28.8 ± 18.3	0.867
Total protein, g/day	51.1 ± 25.5	51.4 ± 25.0	51.6 ± 26.2	0.986
Cholesterol, mg/day	159.9 ± 119.7	181.4 ± 158.8	186.2 ± 149.5	0.245
Fiber, g/day	3.8 ± 2.3	3.6 ± 2.0	3.9 ± 2.4	0.421
Folate, mg/day	152.1 ± 114.1	145.2 ± 88.2	157.3 ± 104.4	0.451
Total phosphorus, mg/day	737.4 ± 332.8	739.8 ± 340.7	745.1 ± 361.5	0.980
Retinol, ug/day	65.6 ± 48.7	75.4 ± 62.2	72.8 ± 59.5	0.249
Total sodium, mg/day	1,685.8 ± 1,076.7	1,684.0 ± 1,061.5	1,634.4 ± 1,009.7	0.879
Total Vitamin A, ug/day	310.3 ± 234.2	327.0 ± 226.2	353.9 ± 289.0	0.297
Vitamin B1, mg/day	0.9 ± 0.4	0.9 ± 0.5	0.9 ± 0.4	0.975
Vitamin C, mg/day	67.9 ± 59.2	65.3 ± 49.0	70.6 ± 61.2	0.615
Total calcium, mg/day	305.0 ± 200.3	303.4 ± 184.4	316.0 ± 218.1	0.806

Data are presented as mean ± SD.
p values were calculated using analysis of variance (ANOVA).

Table S2. Statistical significance among three groups by PSA using distance matrices for beta-diversity.

Diversity index	PERMANOVA		Pairwise PERMANOVA					
	G1 vs. G2 vs. G3		G1 vs. G2		G1 vs. G3		G2 vs. G3	
	Pseudo-F	p value	Pseudo-F	p value	Pseudo-F	p value	Pseudo-F	p value
Unweighted UniFrac distance	1.271	0.149	1.407	0.099	0.569	0.964	1.606	0.067
Weighted UniFrac distance	0.484	0.853	0.536	0.722	0.382	0.847	0.501	0.733
Jaccard distance	1.081	0.139	1.121	0.148	0.947	0.684	1.128	0.147
Bray-Curtis dissimilarity	0.984	0.487	1.066	0.292	0.837	0.816	1.000	0.453

Statistics were calculated using pairwise PERMANOVA with 999 permutations.

Table S3. Comparison of taxonomic composition of gut microbiota among PSA groups.

Model	ANCOM (G2 vs. G3)	MaAsLin (G2 vs. G3)		MaAsLin (G2 vs. G1)	
**Taxa[a]**	**W[b]**	**coefficient[c]**	**p value**	**coefficient[c]**	**p value**
p_Proteobacteria; c_Gammaproteobacteria;o_Enterobacterales;f_Enterobacteriaceae;g_Escherichia-Shigella	221	0.34	5.5.E-04**	0.13	1.7.E-01
p_Firmicutes;c_Negativicutes;o_Veillonellales-Selenomonadales;f_Selenomonadaceae;g_Megamonas	212	-0.26	1.2.E-02*	-0.11	2.9.E-01

p_: phylum; c_: class; o_: order; f_: family; g_: genus; s_: species.

[a] Total number of genera, 251

[b] W = X for taxon k, then H0k is rejected X times. The W statistic for the significantly different taxa relative to more than 80% of the other taxa at each taxon level are represented in bold.

[c] Coefficients for log-transformed relative abundance of each taxon in the linear model adjusted for age, sex, and BMI using MaAsLin pairwise between two groups. * p <0.05, **p <0.01

The G2 group was used as the reference group in the regression model.

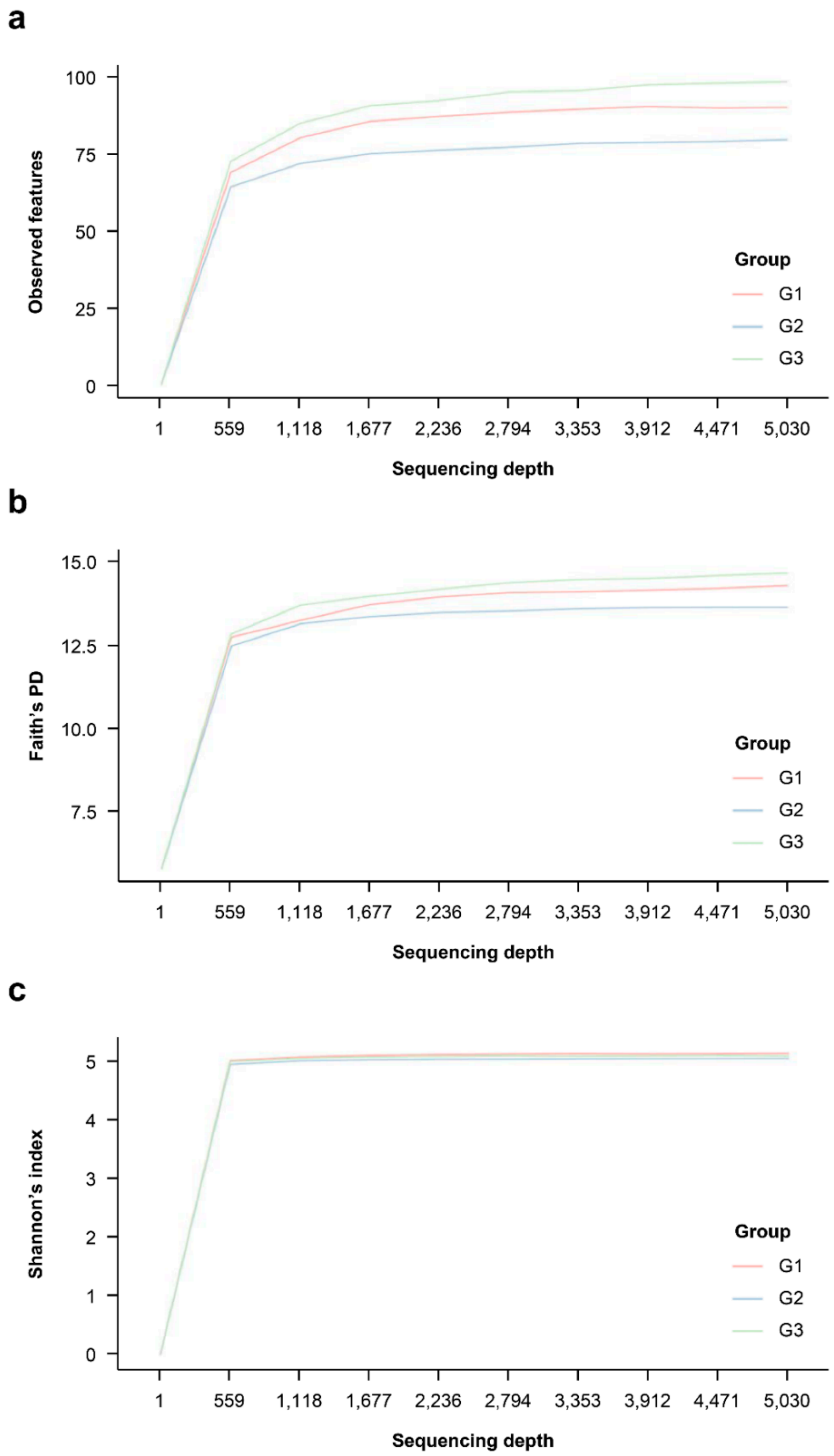

Figure S1. Rarefaction plots based on alpha diversity metrics. (a) The number of observed features, (b) Faith's PD, and (c) Shannon's diversity indicated that 5,030 sequences per sample are sufficient for capturing the alpha diversity of microbial communities in the tree groups by PSA. The x-axis shows the number of sequences per sample. Rarefaction curves construction (10 replicates/depth) were performed using the “diversity alpha-rarefaction" plugin QIIME2.

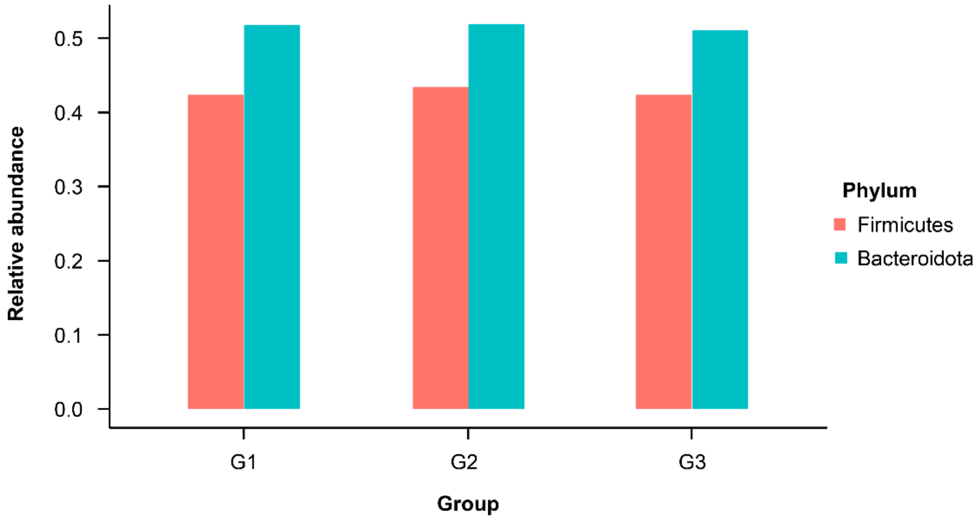

Figure S2. Mean relative abundance of Firmicutes and Bacteroidetes among PSA groups.
